# A Hydrodynamic Model for Measuring Fluid Density and Viscosity by Using Quartz Tuning Forks

**DOI:** 10.3390/s20010198

**Published:** 2019-12-29

**Authors:** Mi Zhang, Dehua Chen, Xiao He, Xiuming Wang

**Affiliations:** 1 State Key Laboratory of Acoustics, Institute of Acoustics, Chinese Academy of Sciences, Beijing 100190, China; zhangmi@mail.ioa.ac.cn (M.Z.);; 2 University of Chinese Academy of Sciences, Beijing 100049, China; 3 Beijing Engineering Researcher Center of Sea Deep Drilling and Exploration, Institute of Acoustics, Chinese Academy of Sciences, Beijing 100190, China

**Keywords:** density sensor, viscosity sensor, quartz tuning fork, sensitivity analysis

## Abstract

A hydrodynamic model of using quartz tuning forks (QTFs) for density and viscosity sensing, by measuring the resonance frequency and quality factor, has been established based on the cantilever beam theory applied to the atomic force microscope (AFM). Two examples are presented to verify the usability of this model. Then, the Sobol index method is chosen for explaining quantitatively how the resonance frequency and quality factor of the QTFs are affected by the fluid density and viscosity, respectively. The results show that the relative mean square error in viscosity of the eight solutions evaluated by the hydrodynamic model is reduced by an order of magnitude comparing with Butterworth–Van Dyke equivalent circuit method. When the measured resonance frequency and quality factor of the QTFs vary from 25,800–26,100 Hz and 28–41, the sensitivities of the quality factor affected by the fluid density increase. This model provides an idea for improving the accuracy of fluid component recognition in real time, and lays a foundation for the application of miniaturized and cost-effective downhole fluid density and viscosity sensors.

## 1. Introduction

The measurement of fluid density and viscosity is essential in petrochemical, food, automotive and textile industries. In automotive industry, on-site monitoring viscosity of lubricants is a key indicator for tracking their performance [1]. In the wine industry, on-line density monitoring during fermentation is also crucial [2,3]. For more than a decade, tuning fork sensors have been used to detect the density and viscosity of fluids in complex downhole environments [4,5,6,7,8,9]. The density and viscosity of reservoir fluids are the main parameters for fluid identification [10], determining fluid composition and dividing the oil–water interface [11], which can help oilfield companies determine reservoir recovery rates, predict productivity and guide reservoir development strategies. On the whole, fluid density and viscosity (D–V) sensors are becoming more widely used.

The Butterworth–Van Dyke equivalent circuit is an important method to study the measurement of liquid density and viscosity [12,13,14,15,16,17]. In this method, a tuning fork resonator can be seen as a series connection of resistor, capacitor and inductor. The interaction of the resonator with surrounding fluid is modeled by an additional contribution to the impedance. The fluid density and viscosity can be obtained by measuring admittance, resonance frequency or other equivalent parameters. However, the evaluated error of viscosity is too large [18,19,20,21]. For example, the maximum relative errors of liquid viscosity and density measured by J. Toledo et al. [18] with a quartz tuning fork were 26.32% and 3.16%, respectively. These errors of liquid viscosity and density by Liu, Y. et al. [19] with lithium niobate tuning fork were 10.48% and 2.89%, respectively. Although it is a physical fact for tuning fork sensors that the error on density is smaller than that on viscosity [22], we can still reduce the viscosity error to meet the measurement requirements.

Tuning fork sensors, microcantilever beam, AlN resonator, torsional resonators and so on, can all be used as sensitive components for the D–V sensor [23,24,25]. On the one hand, quartz tuning forks (QTFs) studied in this paper have high Curie temperature, good stability and high accuracy, which is suitable for downhole high temperature and high pressure environment, and, on the other hand, a millimeter-sized QTF can be integrated in small-scale measurement platforms for downhole deployment. In addition, the QTFs are low-cost and commercially used as frequency standards in watches, working at 32.768 kHz. The QTF is widely used in gas sensing based on photoacoustic spectroscopy and photothermal spectroscopy [26,27,28], and scanning probe microscopy applications such as atomic force microscopy (AFM) [29,30,31] and near-field scanning optical microscopy (NSOM) [32,33,34]. In order to estimate the density and viscosity of a liquid at the same time, the hydrodynamic model based on the work of Sader, J.E. [35] for the atomic force microscope (AFM) is established, and two examples are verified by using this model.

## 2. Hydrodynamic Model

A quartz tuning fork oscillator is composed of two symmetrical, clamped-free tuning fork prongs, which are connected to the central holder. Assume that the cross section of tuning fork prong is uniform over its entire length and the tuning fork is isotropic and homogeneous. Each prong can be individually considered as a clamped-free cantilever beam, and the symmetrical prongs of QTF reduce the number of possible modes. Since the length *L* of each prong of the tuning fork is much larger than its width *l* and thickness *e*, according to the beam theory of Euler–Bernoulli, the governing differential equation for the deflection w(x,t) of the beam [35,36,37] is given by

(1) $$EI\frac{\partial^{4}w(x,t)}{\partial x^{4}}+\rho_{q}el\frac{\partial^{2}w(x,t)}{\partial t^{2}}=F(x,t),$$

where *I* is the moment of inertia, *E* and *ρ_q_* respectively represent the young’s modulus, density of the beam. When vibrating in fluid, the quartz tuning fork per unit length is subjected to an external force

(2) $$F(x,t)=F_{ex}(x,t)+F_{hydro}(x,t),$$

where $F_{ex}(x,t)$ and $F_{hydro}(x,t)$ represent the applied excitation and hydrodynamic force, respectively. We suppose that the fluid is incompressible with a homogeneous density *ρ* and dynamic viscosity *μ*. Taking the Fourier transform of Equation (1), the governing equation can be expressed as

(3) $$[EI\frac{\partial^{4}}{\partial x^{4}}-\omega^{2}\rho_{q}el]W(x,\omega )-F_{hydro}(x,\omega )=F_{ex}(x,\omega ),$$

where the hydrodynamic force per unit length on oscillating cylinder is defined as follows

(4) $$F_{hydro}(x,\omega )=\frac{\pi }{4}\rho \omega^{2}l^{2}\Gamma_{hydro}(\omega )W(x,\omega ).$$

The constant *l* is diameter for a circular cylinder, whereas it is the width for a rectangular beam as well [34]. The exact analytical result of dimensionless hydrodynamic function [38,39]

(5) Γhydro(ω)=1+4K1(jRe)jReK0(jRe),

where *K*_0_ and *K*_1_ are the zero-order and the first-order modified Bessel functions of the second kind, Reynolds number Re=ρωl24μ. The hydrodynamic function can be separated into real part and imaginary part

(6) $$\Gamma_{hydro}(\omega )=\Gamma_{{}_{hydro}}^{r}(\omega )-j\Gamma_{{}_{hydro}}^{i}(\omega ),$$

where the coefficient $\Gamma_{hydro}^{r}(\omega )$ is related to the added mass of the beam, and $\Gamma_{hydro}^{i}(\omega )$ corresponds to the damping coefficient. Substituting Equations (6) and (4) into (3), we find

(7) $$\left[EI\frac{\partial^{4}}{\partial x^{4}}-\omega^{2}\rho_{q}el(1+\frac{\pi \rho l}{4\rho_{q}e}(\Gamma_{hydro}^{r}(\omega )-j\Gamma_{hydro}^{i}(\omega )))]W(x,\omega )=F_{ex}(x,\omega \right).$$

**Figure 1:**
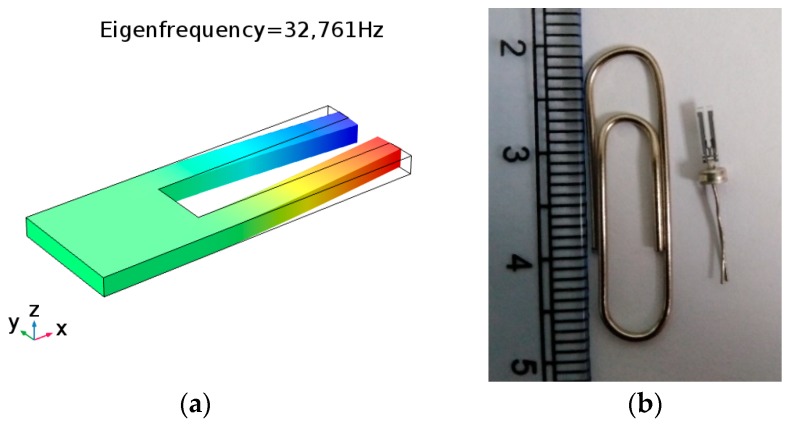
(**a**) The simulation diagram of the first in-plane mode of quartz tuning fork and (**b**) photograph of a millimeter-sized quartz tuning fork.

This work only considers the first in-plane tuning fork mode, as shown in Figure 1. Noting that in the absence of dissipative effects [40], the angular resonance frequency *ω*_1_ of the first vibration mode for the immersed cantilever beam is obtained from Equation (7).

(8) $$\omega_{0}^{2}=\omega_{1}^{2}(1+\frac{\pi l}{4\rho_{q}e}\rho \Gamma_{hydro}^{r}(\omega_{1})),$$

where $\omega_{0}=\frac{\alpha_{1}^{2}l}{2L^{2}}\sqrt{\frac{E}{3\rho_{q}}}$ is the fundamental angular resonance frequency of the beam in vacuum. Depending on the beam boundary condition of clamped-free case, *α*_1_ = 1.875 is the first positive root of $1+\cos(\alpha_{n})\cosh(\alpha_{n})=0$. Quality factor of the first in-plane tuning fork mode for vibration in an incompressible fluid is

(9) $$Q_{1}=\frac{\frac{4\rho_{q}e}{\pi \rho l}+\Gamma_{hydro}^{r}(\omega_{1})}{\Gamma_{hydro}^{i}(\omega_{1})}.$$

**Figure 2:**
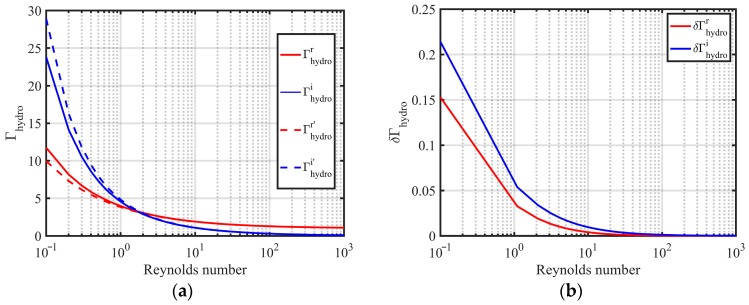
(**a**) Comparison of analytical solutions and approximate solutions between the real part and the imaginary part of hydrodynamic function and (**b**) the relative errors between the approximate solutions and the analytical solutions of the real part and the imaginary part of the hydrodynamic function.

The hydrodynamic function can be simplified by using the progressive expansion of Bessel function [41], as shown in Figure 2. The dimensionless hydrodynamic function $\Gamma_{hydro}(\omega )$ is simplified to

(10) Γhydro(ω)=1+22Re−j(22Re+2Re).

In Equation (10), the approximation values Γhydror′(ω)=1+22Re and Γhydroi′(ω)=22Re+2Re. In Figure 2, when the Reynolds number is greater than 1, the relative errors between the approximation values and the real values of the hydrodynamic function are less than 5%.

Thus, the resonance frequency and quality factor of the first in-plane mode of tuning fork vibrating in an incompressible fluid can be simplified to

(11) $$\omega_{0}^{2}=\omega_{1}^{2}(1+\frac{\pi l}{4\rho_{q}e}\rho \Gamma_{hydro}^{r^{\prime }}(\omega_{1})),$$

and

(12) $$Q_{1}=\frac{\frac{4\rho_{q}e}{\pi \rho l}+\Gamma_{hydro}^{r^{\prime }}(\omega_{1})}{\Gamma_{hydro}^{i^{\prime }}(\omega_{1})}.$$

If the quality factor is far greater than 1, dissipative effects in the fluid can be ignored. In practice, we did not consider dissipative effects when the quality factor of the tuning fork resonator is greater than 10. By rearranging Equations (11) and (12), the fluid mass density and viscosity can be rewritten into the following Equations

(13) $$A\sqrt{\frac{\mu \rho }{\omega_{1}}}+B\frac{\mu }{\omega_{1}}=\frac{1}{Q_{1}}\frac{\omega_{0}^{2}}{\omega_{1}^{2}},$$

(14) $$A\sqrt{\frac{\mu \rho }{\omega_{1}}}+C\rho =\frac{\omega_{0}^{2}}{\omega_{1}^{2}}-1,$$

where *A*, *B* and *C* are constants.

Generally, the resonance frequency and quality factor of QTF sensor are affected by fluid density and viscosity. The hydrodynamic model, as shown in Equations (13) and (14), is used to reverse the density and viscosity of the fluid according to the resonance frequency and quality factor of the QTF.

## 3. Model Validation

In the hydrodynamic model, the quartz tuning fork D–V sensor needs to be calibrated before the actual measurements. By measuring the resonance frequency and quality factor of two liquids with known density and viscosity, the calibration parameters *A*, *B* and *C* can be obtained from Equations (13) and (14). Using the resonance frequency and quality factor of quartz tuning fork reported by J. Toledo et al. [18] and Voglhuber-Brunnmaier T et al. [42], respectively, to validate the hydrodynamic model, we estimated the density and viscosity values of various liquids after calibration and calculated their relative errors.

### 3.1. Example 1

**Figure 3:**
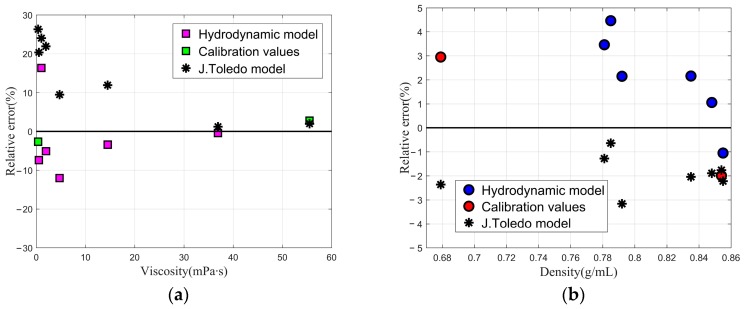
The hydrodynamic model is calibrated by using two liquids (green box for viscosity and red circle for density). The black star represents the results calculated by J. Toledo et al. (**a**) The relative errors of viscosity are verified for the six additional fluids (pink box) and (**b**) the relative errors of density are verified for the six additional fluids (blue circle).

Referring to the resonance frequency and quality factor of quartz tuning fork reported by J. Toledo et al. [18], we selected heptane and N35 with known density and viscosity for calibration to get the values of *A* = 6.3586 m^2^/kg, *B* = −40667 m/kg and *C* = 2.8788 × 10^−4^ m^3^/kg in the viscosity range of 0.38–55.52 mPa∙s and density range of 0.679–0.854 g/mL. Table 1 shows the estimated values of density and viscosity for different liquids deduced from the hydrodynamic model. Figure 3a,b show relative errors of density and viscosity for the different liquids.

From Table 1, the relative error of ethanol viscosity evaluated by hydrodynamic model is 16.35%. The reason for large viscosity error of ethanol is that the quality factor of the tuning fork sensor in ethanol measured by J. Toledo et al. [18] is low. To comprehensively compare the accuracy of hydrodynamic model and Butterworth–Van Dyke equivalent circuit used in [18], the relative mean square error (MSE) was expressed as the average of the square of relative error of the data. The smaller value of relative MSE, the better accuracy of the prediction model to describe the experimental data. The relative MSE for Butterworth–Van Dyke equivalent circuit is 0.03 for liquid viscosity and 0.00042 for liquid density in [18]. However, the relative MSE for the hydrodynamic model is 0.0067 for liquid viscosity and 0.0007 for liquid density. Compared with the work of J. Toledo, the relative mean square error in viscosity of the eight solutions evaluated by the hydrodynamic model is reduced by an order of magnitude.

### 3.2. Example 2

**Figure 4:**
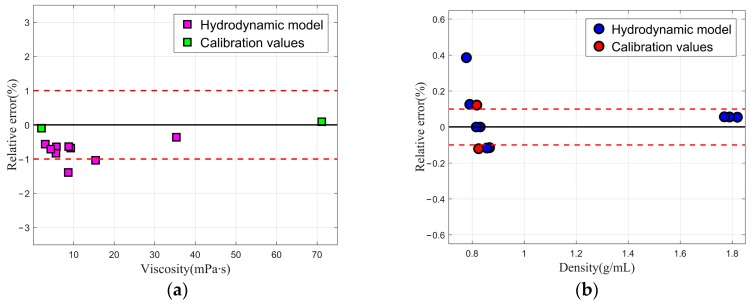
The hydrodynamic model is calibrated by using two liquids (green box for viscosity and red circle for density). (**a**) The relative errors of viscosity are verified for the nine additional fluids (pink box) and the relative errors of viscosity reported by Voglhuber-Brunnmaier T et al. (between two red dotted lines) and (**b**) the relative errors of density are verified for the nine additional fluids (blue circle) and the relative errors of density reported by Voglhuber-Brunnmaier T et al. (between two red dotted lines).

As in Voglhuber-Brunnmaier T et al. [42], we selected sample Liquid 4 and sample Liquid 5 with known densities and viscosities for calibration in the viscosity range of 2–71.149 mPa∙s and density range of 0.778–1.820 g/mL. Since the tuning forks mentioned in [18] and [42] are different in sizes, the values of the calibration parameters *A* = 5.6337 m^2^/kg, *B* = 28,133 m/kg and *C* = 3.0547 × 10^−4^ m^3^/kg are also different. The estimated values of density and viscosity for the various liquids deduced from the hydrodynamic model are shown in Table 2. Figure 4a,b show relative errors of density and viscosity for the eleven different liquids.

The measuring precision calculated by the hydrodynamic model is close to that reported by Voglhuber-Brunnmaier T et al. [42], which achieves accuracies in the range of 1% in viscosity and 0.1% in mass density. Voglhuber-Brunnmaier T et al. [42] uses a generalized reduced order model, which is the most accurate method for measuring fluid density and viscosity with tuning fork sensors at present. As shown in Figure 4, the relative errors of the eleven liquids’ viscosity and density were almost within ±1% and ±0.2%, respectively, by using the hydrodynamic model. In general, the hydrodynamic model can evaluate fluid density and viscosity with satisfied accuracy. However, the hydrodynamic model requires less coefficients, which saves computing time. Therefore, this simplified model proposed in this study was reliable and efficient.

## 4. Sensitivity Analysis

For resonant D–V sensors, absolute sensitivities cannot be applicable. To globally analyze the sensitivity of resonant tuning fork D–V sensors, the Sobol index method [43] was applied to the hydrodynamic model to quantitatively evaluate the influence of quality factor or resonance frequency affected by viscosity or density. The characteristic of the Sobol index method is that all parameters can change simultaneously in the whole investigable range of parameters. Moreover, the nonlinear and non-monotone models can be studied and analyzed using the Sobol index method. The core idea of the Sobol index method is to calculate the influence of the variance of each input parameter on the total output variance, and then the importance of parameters and their interaction effects can be analyzed. For a given model $Y=f(X_{1},X_{2},\ldots ,X_{k})$, the first-order sensitivity indices

(15) $$S_{i}=\frac{V(E(Y|X_{i}))}{V(Y)},i=1,2,\ldots ,k,$$

where *V*(E(Y|*X_i_*)) refers to the variance of the conditional expectation within the effective range of the variable *X_i_*, and *V*(Y) is the total variance of the output. Total-effect sensitivity indices

(16) $$S_{Ti}=1-\frac{V(E(Y|X_{∼i}))}{V(Y)},i=1,2,\ldots ,k,$$

where $X_{∼i}$ are variables other than the variable *X_i_*. *V*(E(Y|*X*_~*i*_)) is the variance of the mean value of Y for all variables other than the variable *X_i_* within the effective range. The total-order sensitivity indices of variable *X_i_* include not only the first-order sensitivity indices of variable *X_i_*, but also the interaction effects between variable *X_i_* and other variables.

**Figure 5:**
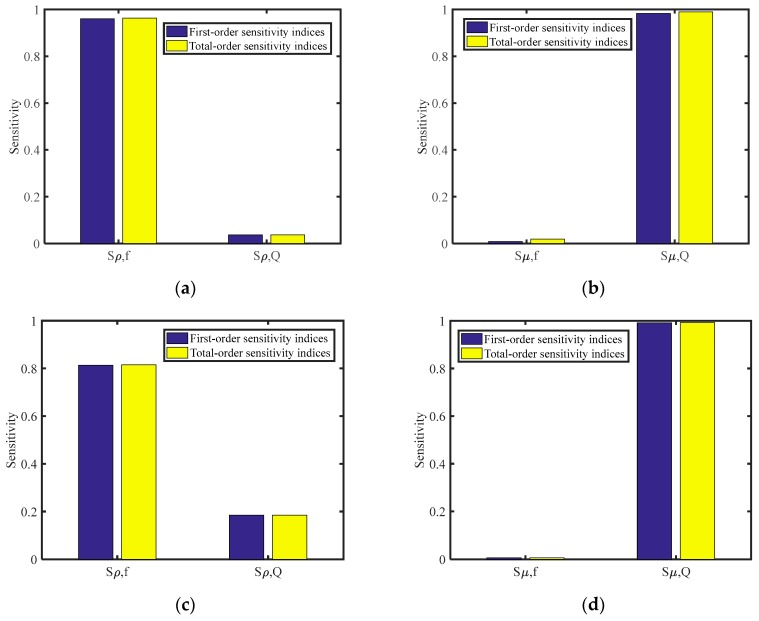
The first- and total-order sensitivity indices for the resonance frequency and quality factor affected by density and viscosity. (**a**) The first- and total-order sensitivity indices affected by density for the resonance frequency range of 25,800–29,810 Hz and quality factor range of 11–165; (**b**) the first- and total-order sensitivity indices affected by viscosity for the resonance frequency range of 25,800–29,810 Hz and quality factor range of 11–165; (**c**) the first- and total-order sensitivity indices affected by density for the resonance frequency range of 25,800–26,100 Hz and quality factor range of 28–41; and (**d**) the first- and total-order sensitivity indices affected by viscosity for the resonance frequency range of 25,800–26,100 Hz and quality factor range of 28–41.

A sensitivity analysis of the Sobol index method was used to determine the influence of various parameters on the output of hydrodynamic model. According to Equations (13) and (14), the model has two input variables: resonance frequency and quality factor, and two output variables: density and viscosity. Assume that the lower limit of resonance frequency was 25,800 Hz, and the upper limit was 29,810 Hz. The range of the quality factor varied from 11 to 165. We sampled the above two input parameters with a Sobol sequence and selected a sampling point of 10,000. The influence of resonance frequency and quality factor on the calculated results of density and viscosity were obtained. The first-order sensitivity indices and the total-order sensitivity indices for the inputs are shown in Figure 5.

In Figure 5a, the first- and total-order sensitivity indices for resonance frequency affected by density were much higher than those for quality factor affected by density. It shows that the change of resonance frequency was more sensitive to the density than to quality factor within the range of the QTF measurement. In Figure 5b, the change of quality factor was more sensitive to the viscosity than to density. 

We observed that the density of sample Liquid 9, 10 and 11 was much higher than the density of other liquids. Assume that the resonance frequency and quality factor vary from 25,800–26,100 Hz and 28–41, respectively. The sensitivity analysis was calculated again using the Sobol index method. In Figure 5c,d, the first- and total-order sensitivity indices for the resonance frequency affected by density decrease, however, the first- and total-order sensitivity indices for the quality factor affected by density increase. In other words, when the density of the solution is very high, it will not only reduce the resonance frequency of the QTF, but also the quality factor of the QTF. It indicates that comparing with the quality factor of the QTF in two liquids with similar viscosities and large differences in density, one liquid with higher density has a lower quality factor of the QTF. This explains why the measured quality factor of the QTF in sample Liquid 10, with reported density 1.79 g/mL and viscosity 5.653 mPa∙s, was smaller than that of the QTF in sample Liquid 8, with reported density 0.778 g/mL and viscosity 5.75 mPa∙s.

It is generally believed that the resonance frequency of the tuning fork sensor decreases as the density of liquid increase, and its quality factor decreases as the viscosity of liquid increases. However, when the density of one solution is very high, the quality factor affected by density works, such as sample Liquid 8 versus sample Liquid 10 in Example 2, sample Liquid 7 versus sample Liquid 9 in Example 2, etc.

## 5. Sensitivity to Temperature

**Figure 6:**
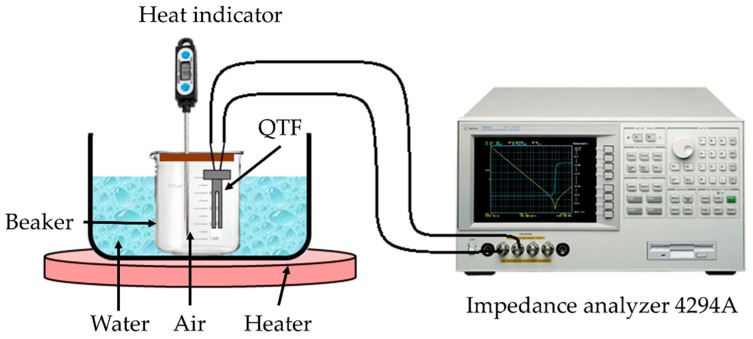
High temperature measurement setup of the quartz tuning fork (QTF) characteristics.

The downhole temperature environment affects not only the density and viscosity of the fluid, but also the resonant frequency of the QTF. To calculate the density and viscosity of the fluid as accurately as possible, it is necessary to analyze the effect of temperature on quartz tuning fork. The resonance frequency of the QTF sensor was evaluated in temperature range from 40 to 70 °C in air. The temperature of the constant temperature bath was adjusted every 10 °C, and the output signal of the sensor was detected and analyzed by a high precision Agilent 4294A impedance analyzer, see Figure 6.

**Figure 7:**
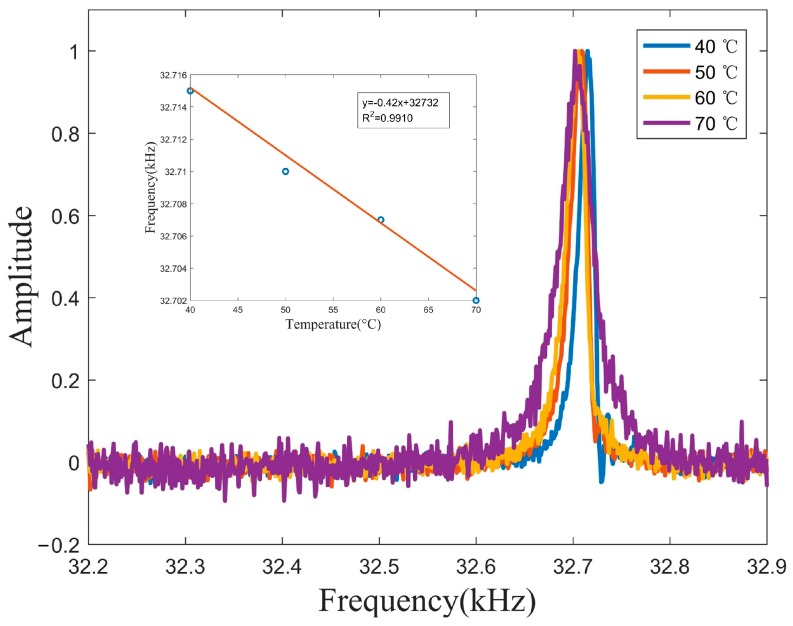
Measured frequency responses of QTF in different temperature and the evaluated dependencies of resonance frequencies on temperature.

The dependence of the QTF’s fundamental resonance frequency to temperature was shown in Figure 7. The evaluated dependencies of resonance frequencies on temperature ranging from 40 to 70 °C in air were −0.42 Hz/°C. The measured quality factors of Cannon S20 in Example 1 and sample Liquid 6 in Example 2 are almost the same and the measured resonance frequency differed by 54 Hz. However, the reported densities were 0.816 g/mL and 0.855 g/mL, respectively. Therefore, before measuring the density and viscosity of fluid with the QTF in different temperature environment, the QTF needs to be temperature calibrated, such as using circuit design or formulating temperature dependent model.

## 6. Discussion and Conclusions

The hydrodynamic model presented in this paper was derived and simplified from the beam theory of Euler–Bernoulli. This model was validated to estimate the density and viscosity of the fluids when the measured quality factor of the tuning fork exceeds 10. Compared with Butterworth–Van Dyke equivalent circuit method, the relative mean square error in viscosity of the eight solutions evaluated by this simplified model was reduced by an order of magnitude. In addition, for the tuning fork resonant sensors, the sensitivity indices of the resonance frequency to viscosity increased when the measured resonance frequency and quality factor of the QTFs varied from 25,800–26,100 Hz and 28–41. It explains why the liquid, with very high density, will not only reduce the resonance frequency of the QTFs, but the quality factor of the QTFs. The temperature dependent model and experiments of various liquids at high temperature environment will be studied next.

## Figures and Tables

**Table 1:** Resonance frequency and quality factor are measured and the density and viscosity of eight samples are calculated.

Fluid	Tuning Fork		Evaluated Values		Relative Deviations	
	*Q*	f1 (Hz)	*μ* (mPa∙s)	*ρ* (g/mL)	δ*μ* (%)	δ*ρ* (%)
Heptane	163.76	29,804.5	0.37	0.699	−2.63	2.95
Methanol	133.87	29,400.0	0.50	0.809	−7.41	2.15
Ethanol	86.18	29,299.5	1.21	0.820	16.35	4.46
2-Propanol	70.45	29,298.0	1.86	0.808	−5.10	3.46
D5	46.92	29,033.9	4.17	0.853	−12.03	2.16
N10	26.68	28,756.4	14.01	0.857	−3.38	1.06
S20	17.87	28,460.0	35.37	0.846	−4.20	−1.05
N35	14.75	28,253.0	57.05	0.837	2.76	−1.99

**Table 2:** Resonance frequency and quality factor are measured and the density and viscosity of eleven samples are calculated.

Sample	Tuning Fork		Evaluated Values		Relative Deviations	
	*Q*	f1 (Hz)	*μ* (mPa∙s)	*ρ* (g/mL)	δ*μ* (%)	δ*ρ* (%)
1	25.8858	28,599	15.270	0.866	−1.037	−0.115
2	34.8459	28,769	8.575	0.856	−1.391	−0.117
3	60.0072	29,025	2.986	0.831	−0.566	0
4	72.6525	29,108	2.064	0.819	−0.097	0.122
5	11.8873	28,157	71.213	0.824	0.090	−0.121
6	17.1759	28,514	35.222	0.816	−0.362	0
7	34.4446	28,990	9.218	0.792	−0.679	0.126
8	44.1299	29,119	5.713	0.781	−0.643	0.386
9	28.1085	25,808	8.747	1.821	−0.636	0.055
10	35.2417	25,977	5.606	1.791	−0.831	0.056
11	40.1609	26,073	4.339	1.771	−0.709	0.057
